# Leveraging Cholesterol-Functionalized Cyclodextrin Nanosponges for Enhanced Drug Delivery in Cancer Cells

**DOI:** 10.3390/ijms26031213

**Published:** 2025-01-30

**Authors:** Ilona Krabicová, Yousef Khazaei Monfared, Fabrizio Caldera, Mohammad Mahmoudian, Christopher Hobbs, Rosangela Santalucia, Silvia Lucia Appleton, Adrián Matencio, Parvin Zakeri-Milani, Francesco Trotta

**Affiliations:** 1 Department of Chemistry, Faculty of Science, Humanities and Education, Technical University of Liberec, 461 17 Liberec, Czech Republic; 2 Department of Chemistry, University of Turin, 10125 Turin, Italy; ykhazaeimonfared@mgh.harvard.edu (Y.K.M.); fabrizio.caldera@unito.it (F.C.); rosangela.santalucia@unito.it (R.S.); adrian.matencioduran@unito.it (A.M.); 3 Division of Nuclear Medicine and Molecular Imaging, Department of Radiology, Massachusetts General Hospital, Harvard Medical School, Boston, MA 02114, USA; 4 NIS Interdepartmental Centre, 10125 Turin, Italy; 5 Department of Oncology, University of Torino, 10060 Candiolo, Italy; mahmoodian.nano@gmail.com; 6 Candiolo Cancer Institute, FPO-IRCCS, 10060 Candiolo, Italy; 7 Department of Nanochemistry, Institute of Nanomaterials, Advanced Technology and Innovation, Technical University of Liberec, 461 17 Liberec, Czech Republic; christopher.hobbs@tul.cz; 8 Institute of Genetic and Biomedical Research, 20139 Milan, Italy; silvialuciaappleton93@gmail.com; 9 Liver and Gastrointestinal Diseases Research Centre and Faculty of Pharmacy, Tabriz University of Medical Sciences, 5166614756 Tabriz, Iran; pzakeri@tbzmed.ac.ir

**Keywords:** cholesterol, nanosponge, drug delivery, crosslinked polymer, functionalization

## Abstract

Cholesterol, the essential building block of cellular membranes, has proven to be a useful tool for increasing the biocompatibility and bioavailability of drug delivery systems in cancer treatment. Resveratrol, a natural polyphenolic compound with promising anticancer properties, faces significant limitations due to its low solubility and bioavailability, hindering its clinical utility. Therefore, in the present study, we aimed to design cholesterol-functionalized cyclodextrin nanosponges (Chol-NSs) with a tunable cholesterol content to optimize resveratrol encapsulation and delivery. Both formulations, free carbonyl diimidazole (CDI) NSs and functionalized Chol-NSs, demonstrated high drug loading and encapsulation efficiency. In vitro experiments revealed that cholesterol incorporation significantly improved the cellular uptake of nanocarriers and potentiated the cytotoxic effects of resveratrol on breast cancer cells. Importantly, both free CDI NSs and functionalized Chol-NSs, even at varying cholesterol percentages, demonstrated a safe profile against both fibroblast and breast cancer cell lines. These results indicate that cholesterol-functionalized nanosponges represent a promising platform for the effective and safe delivery of natural compounds in cancer therapy.

## 1. Introduction

Cyclodextrins (CDs), cyclic oligosaccharides, have demonstrated an outstanding capability to entrap molecules in their relatively hydrophobic cavity, leading to the modification of their physico-chemical properties, biological activity, or protection of the guest from degradation [1,2]. Thanks to the large number of hydroxyl groups, CDs can be easily functionalized and hence acquire the desired properties, such as fluorescence, targeting ability, or become a building block of polymeric structure [3,4,5]. On the other hand, the cyclodextrin nanosponges (CD NSs) system, belonging to the class of hyper-crosslinked polymers with CD monomer units as building blocks, has demonstrated remarkable capabilities in delivering anticancer drugs. In both in vitro and in vivo studies, the application of CD NSs has yielded promising results, enhancing the solubility, sustaining release, improving bioavailability, and protecting anticancer drugs from degradation [6,7,8]. In contrast to CDs which form complexes preferably with hydrophobic compounds in aqueous environment, CD NSs are capable of encapsulating both hydrophobic and hydrophilic molecules [9,10,11]. Hence, due to the easy modifiability of CD NSs as a drug delivery system and their ability to meet the requirements of the specific application, they have shown remarkable efficiency in the treatment of various diseases, including cancers [12,13], diabetes [14], and viral infections [15,16].

Cholesterol is a vital component in the body, serving as a fundamental building block for cellular membranes. It plays a crucial role in hormone metabolism, cell adhesion, and facilitating the exchange of signals between cells and their surrounding environment. Therefore, incorporating cholesteryl modifications into drug carriers can be advantageous for enhancing biocompatibility, bioavailability, and the ability of carriers to penetrate cells [17]. Various studies have shown the potential of cholesteryl-functionalized nanostructures in the realms of cancer treatment [18,19] and gene therapy [20,21]. This modification not only improved the internalization of the carrier into cells but also exhibited good biocompatibility with normal cells, such as fibroblasts and cardiomyocytes. Interestingly, the modification led to an increase in toxicity towards cancer cell lines [18,22].

Amphiphilic cholesteryl–CD derivatives were preferentially synthesized with the specific aim of facilitating their self-assembly to membranes, micelles, or liposomes [23,24,25]. Functionalizing cyclodextrin metal–organic frameworks (CD-MOFs) with cholesterol has demonstrated advantages such as enhanced stability in water while preserving the structure, biocompatibility, and drug inclusion capacity of the CD-MOFs [26]. Another study, conducted by Singh et al., demonstrated that the postfunctionalization of cyclodextrin nanosponges with cholesterol resulted in higher biological activity and increased efficiency in delivering doxorubicin [19].

**Figure 1:**
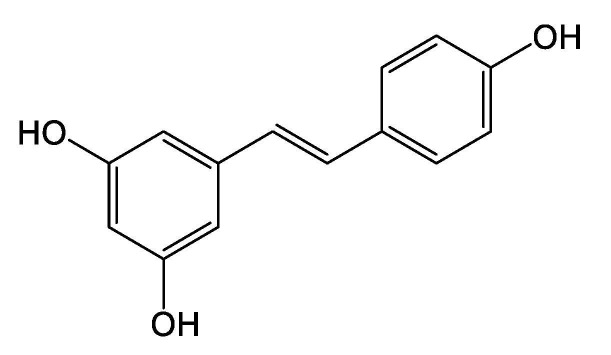
Chemical structure of trans-resveratrol (RSV).

In recent decades, resveratrol (RSV), a natural polyphenol (Figure 1), has shown significant therapeutic potential in the treatment of various diseases, such as periodontal diseases [27], polycystic ovarian syndrome [28] and gynecological cancer [29,30], cerebral ischemic stroke injury [31] and neurodegenerative diseases [32], gastrointestinal cancer [33,34], prostate cancer [35], and breast cancer [36,37,38], because of its antioxidant and anti-inflammatory effects. However, the utility of RSV is hindered by its susceptibility to light and poor water solubility, both of which contribute to its low bioavailability. These limitations pose challenges in formulating effective delivery systems for RSV in order to enhance its clinical efficacy [39]. Therefore, various nano-delivery systems have been used to increase its stability, solubility, and bioavailability, including starch microparticles [39], protein nanofibrils [40], nanostructured lipid carriers [41], gold nanoparticles [42,43], and mesoporous silica nanoparticles [44,45]. Intriguingly, the stability, solubility, and bioavailability of RSV in complexes with CDs [46] and CD NSs [47] have been enhanced [39,48].

As far as our knowledge extends, the literature describes cholesterol-functionalized micelles [22] and polymeric nanoparticles [17,18,20]. However, there is limited research focused on the modification of cyclodextrins and their assemblies, with only a few studies exploring this area [19,26]. Therefore, in the current study, we aimed to prepare and characterize cholesteryl-functionalized cyclodextrin nanosponges with a controllable content of cholesterol in the polymeric structure to increase the cellular uptake and bioavailability of the guest molecule. Additionally, the effect of the lipidic moiety quantity in the structure on the biological interactions was examined.

## 2. Results and Discussion

### 2.1. Synthesis of Modified Chol-NS

The preparation of the nanosponges modified with cholesterol followed a pre-functionalization approach. Initially, the cholesterol moiety was attached to β-cyclodextrin (β-CD), and then the crosslinking process was carried out. The modification of β-CD was based on a previously published method [19]. The final product was analyzed by FTIR, solid-state NMR, and MS to investigate the linkage and ratio between cholesterol and CD.

**Scheme 1:**
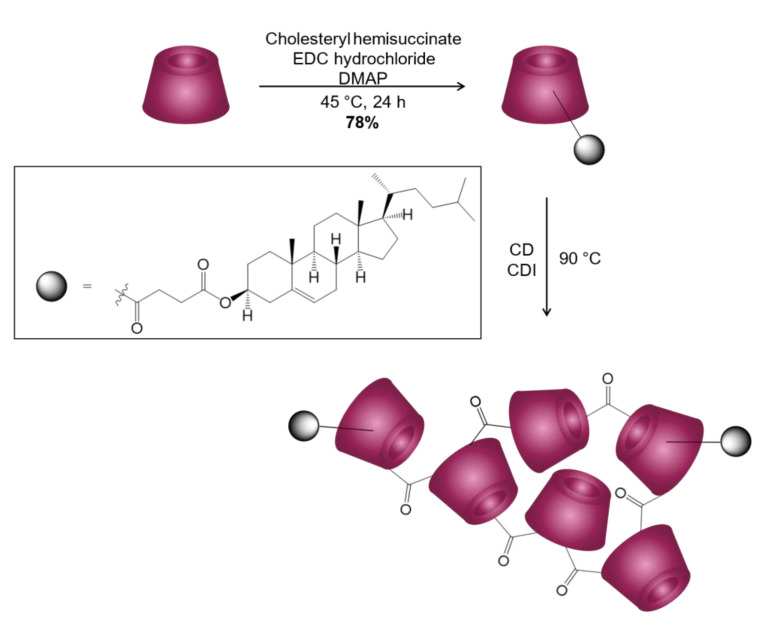
The preparation route of CD nanosponges functionalized with cholesterol.

We have functionalized β-CD and used it for further crosslinking to create novel CD NSs with several advantages (Scheme 1). Firstly, this approach allows for the convenient regulation of the cholesterol content in the end product by blending it with native CD before the crosslinking process in contrast to the postmodification of CD NSs described by Singh et al. [19], where controlling the modification process and the quantity of attached functional groups proved to be less straightforward. The second advantage lies in the flexibility of employing a broad range of crosslinking agents, which helps address issues related to certain types of CD NSs that may not endure the reaction conditions required for their subsequent modification.

The functionalized Chol-β-CD was then used for the NS preparation in the mixture with native β-CD in ratios of 1:9 and 3:7 to see the effect of the cholesterol content on the properties of the material. The results have shown that the cholesterol moiety is an essential factor limiting the crosslinking. The gelation, which is a sign of NS formation, was not observed when the proportion of the Chol-β-CD in the CD mixture was higher than 30%. In addition, the time needed for gelation and the consistency of the gel were strongly dependent on the cholesterol quantity. Crosslinking obstacles might be caused by steric hindrance between functionalized CDs. We expect that our approach might provide a relatively regular distribution of cholesterol in the particles. In the case of the postfunctionalization of CDI nanosponges, we would expect cholesterol to preferentially bind near the particle surface due to low swelling and a short crosslinker. The postmodification of non-swollen particles leads mainly to a surface modification as this location is the most accessible. On the contrary, the swollen state of nanoparticles may enhance the penetration of the functional reagent in the particle interior and enable complete functionalization [49]. Polymeric particles containing cholesterol presented in the literature were commonly designed to form micellar structures, and the final form of the particles was achieved in an aqueous environment [50,51,52]. Since in our demonstrated procedure a mixture of modified and native cyclodextrin was used for the synthesis of the nanosponges and the synthesis was carried out in an organic solvent (specifically DMF), we expect a relatively uniform distribution of cholesterol in the particle mass. This hypothesis could be supported by the limited gelation of the material, which is dependent on the increasing amount of cholesterol in the structure. Obtained materials were subsequently purified and characterized.

### 2.2. FTIR Characterization

The FTIR analysis was performed to provide further information about the structure and to complete the results given by the NMR analysis.

**Figure 2:**
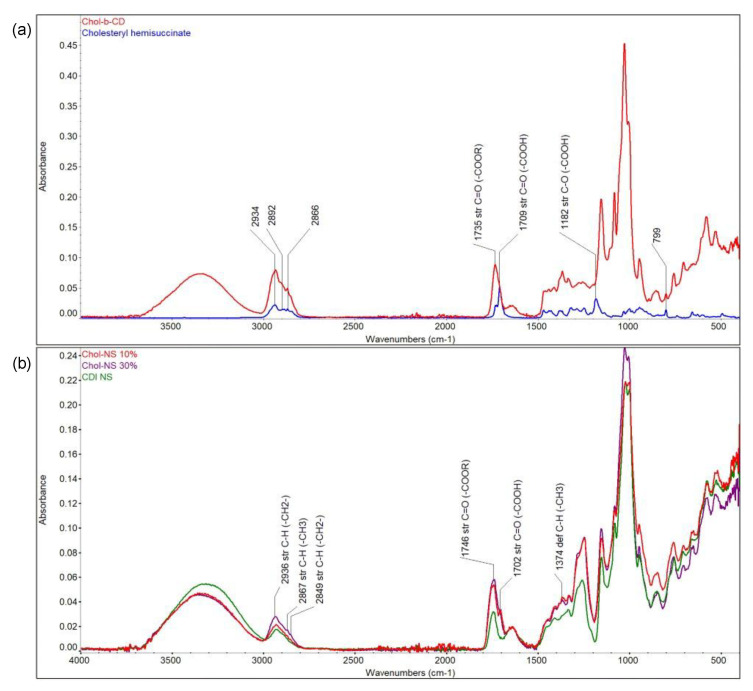
(**a**) FTIR spectra of Chol-β-CD compared with the spectrum of cholesteryl hemisuccinate; (**b**) FTIR spectra of Chol-NS and CDI NS.

The signal of the carboxylic acid of cholesteryl hemisuccinate shifted after binding to CD from 1709 cm^−1^ to 1735 cm^−1^ as the ester bond between the cholesteryl moiety and CD was created (Figure 2a). Moreover, significant changes in other regions could be observed specifically in the intensified stretching vibrations corresponding to -CH_2_- groups of cholesterol between 2800 and 3000 cm^−1^ and decreased the intensity of the cyclodextrin hydroxy group band between 3000 and 3600 cm^−1^ (Figure 2a,b). These observations correspond to the data published in the literature [53].

### 2.3. Mass Spectrometry

For deeper analysis of the modification, FIA-HRMS analysis was performed on the functionalized Chol-β-CD. The spectrum showed signal *m*/*z* = 1603.73 corresponding to monosubstituted Chol-β-CD, and no signs of multiple substitution were found. Interestingly, the study of Shaikh et al. [53] reported that the degree of substitution of their cholesterol-linked CD was equal to 2 and stated that the maximum possible degree of substitution was equal to 3. Thus, modification of the synthetic procedure could possibly lead to the attachment of multiple cholesteryl moieties to the cyclodextrin and subsequently to the increase in the cholesterol content in the structure of the nanosponge.

### 2.4. NMR Characterization

**Figure 3:**
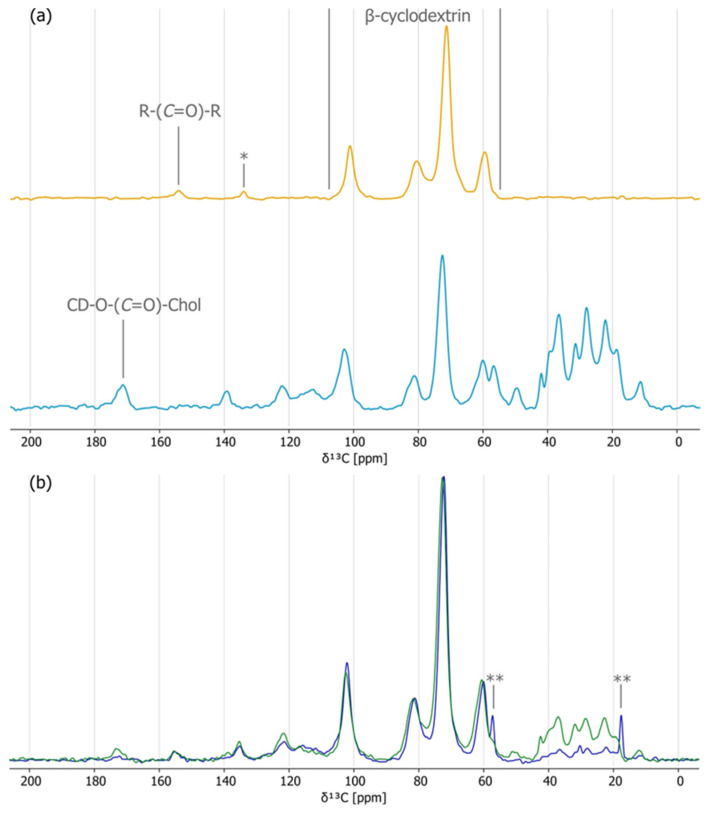
(**a**) Direct pulse ^13^C{^1^H} SS-NMR spectra of CDI NSs (orange) and Chol-β-CD (light blue). (**b**) Direct pulse ^13^C{^1^H} SS-NMR spectra of Chol-NS 10% (dark blue) and Chol-NS 30% (green). * is likely partially reacted CDI with CD, whereby one unreacted imidazole group remains. ** are the residual solvent peaks.

Solid-state NMR measurements of Chol-β-CD and Chol-NS were performed to better understand the structure of the materials and to complete the information given by the FTIR analysis. The ^13^C analysis of the prepared materials revealed that the signals coming from the carbons of the β-CD were between 45 and 130 ppm, the signals of cholesterol were between 20 and 40 ppm, and the signals of the crosslinker were between 145 and 150 ppm. Detailed assignment of the signals is given in the experimental section, and the spectra are shown in Figure 3.

### 2.5. Dynamic Light Scattering (DLS) and Analysis of the Particle Size

The particle size of the nanosponges in aqueous suspension was analyzed with dynamic light scattering. All the materials had particles in the size range between 160 and 220 nm (Table 1). The finest particles were of Chol-NS 30%, with nanosponge having the highest content of cholesterol, which may be related to the structural changes in the gel observed during the crosslinking process. Since the preparation of nanosponges comprises the top-down approach, the limitation of crosslinking observed when increasing the cholesterol content could affect the final particle size.

Whereas the crossing of biological barriers requires very fine particles, cancer cells allow for the entry and accumulation of particles of sizes below 500 nm [54,55]. Although smaller particles can diffuse faster in the tumor, their retention in the tissue might be lower compared to bigger particles [56].

### 2.6. Drug Loading Capacity and Encapsulation Efficiency

The synthesized NSs were subsequently loaded with resveratrol. To quantify the RSV loaded into nanosponges, the active compound was extracted into ethanol. The results of the quantification with HPLC showed that the highest loading capacity as well as encapsulation efficiency showed Chol-NS 10%, whereas Chol-NS 30% and CDI NS showed results similar to each other. The detailed results, for easier readability, are presented in Table 2.

### 2.7. In Vitro Cytotoxicity

**Figure 4:**
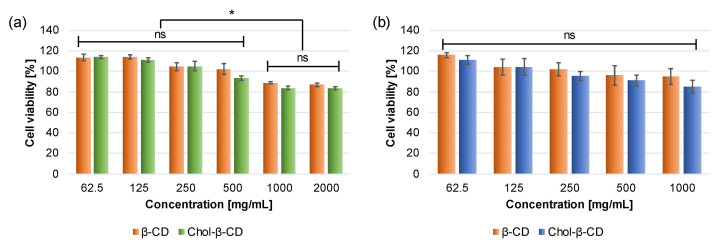
Cell viability of (**a**) fibroblasts and (**b**) breast cancer cells after treatment with blank CD and functionalized with cholesterol. One-way ANOVA: ns, not significant; * *p* < 0.05.

**Figure 5:**
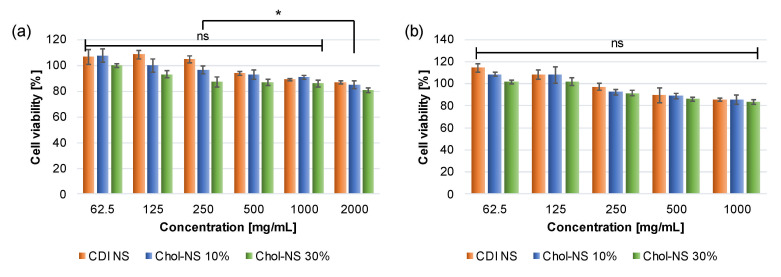
Cell viability of (**a**) fibroblasts and (**b**) breast cancer cells after treatment with suspensions of CDI-NSs and Chol-NSs functionalized with cholesterol. One-way ANOVA: ns, not significant; * *p* < 0.05.

The MTT assay was used to check the cell cytotoxicity of free carriers and loaded with resveratrol. Carbonate cyclodextrin nanosponges have demonstrated a considerable biocompatibility in various studies [57]. Nevertheless, the functionalization can fundamentally change the biological response. Interestingly, both blank β-CD and functionalized Chol-β-CD did not exhibit cytotoxicity against fibroblasts at a concentration of 2 mg/mL and breast cancer cells at 1 mg/mL (Figure 4). Moreover, both blank CDI NSs and functionalized Chol-NSs with varying percentages of cholesterol demonstrated a safe profile against both cell lines even at the highest concentrations (Figure 5). This safe profile for both blank CD and NSs was consistent with the results of other studies [19].

Subsequently, resveratrol (RSV) was loaded on β-CD, CDI-NSs, and NSs functionalized with cholesterol at a ratio of 1:4 (RSV: particles). The aim was to evaluate their impact on the anticancer effectiveness of RSV against MCF-7 cancer cells.

**Figure 6:**
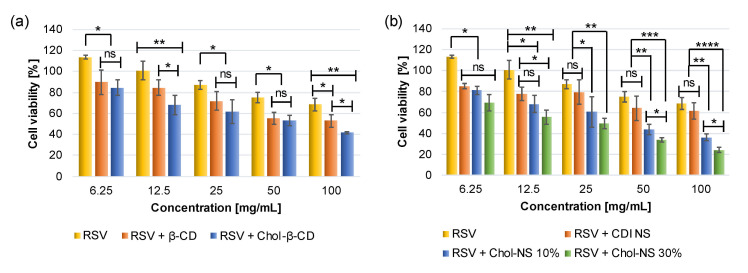
Cell viability of MCF-7 cancer cell line treated with (**a**) the free resveratrol (RSV), loaded on β-CD, and β-CD functionalized with cholesterol; (**b**) RSV loaded on CDI NSs and cholesterol-functionalized Chol-NSs at 10 and 30%. One-way ANOVA: ns, not significant; * *p* < 0.05; ** *p* < 0.01; *** *p* < 0.001; **** *p* < 0.0001.

The results have indicated that the cell cytotoxicity of RSV alone was not considerable against cancer cells even at the highest concentration (0.1 mg/mL), while the RSV loaded into β-CD and, particularly, functionalized with cholesterol enhanced its therapeutic effect against cancer cell in a dose-dependent manner (Figure 6a). Intriguingly, the cytotoxic effect of RSV was significantly enhanced when it was encapsulated with cholesterol-functionalized Chol-NSs, particularly as the percentage of cholesterol increased. This enhancement was peculiarly remarkable at the highest concentration tested (0.1 mg/mL), with the cytotoxicity being 2.5 times greater than that observed with free RSV (Figure 6b). The enhanced efficiency of RSV, a hydrophobic drug, loaded on both CD and NSs observed in this study may be attributed to the functionalization of particles with cholesterol, which led to increased availability through complexation, as well as improved cell internalization. The positive effect of encapsulation on the aqueous solubility and anticancer activity of RSV has been previously shown for both cyclodextrins [58,59] and nanosponges [60].

### 2.8. Cellular Uptake

Based on the evidence demonstrating the increased cell cytotoxicity of RSV loaded on both CD and CDI NSs functionalized with cholesterol compared to free RSV and loaded on non-functionalized particles, we hypothesized that this enhancement in cytotoxicity is associated with cholesterol functionalization, which leads to the increased internalization of both CD and CDI NSs into the cells. Enhancing the cellular uptake by cholesteryl moieties has been shown on various types of nanosystems. However, the dependence of the drug delivery efficiency on the number of cholesteryl groups in the carrier was not described [61].

**Figure 7:**
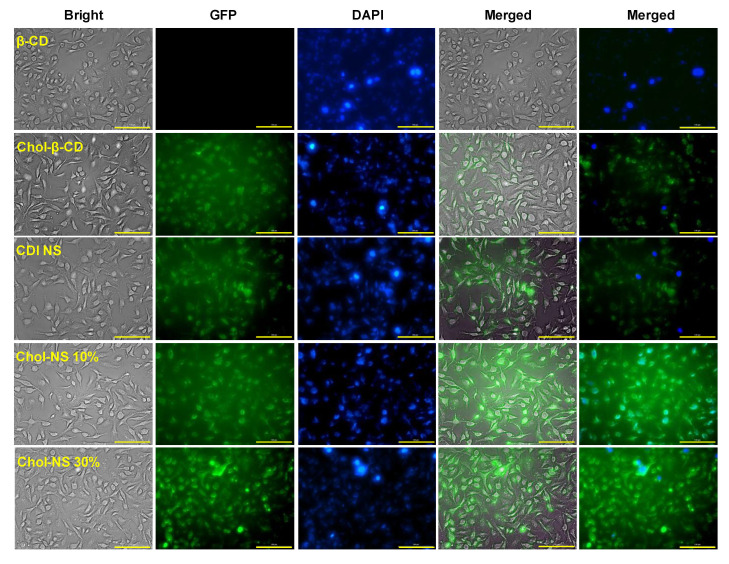
The fluorescence microscopy cellular uptake of β-CD, CDI-NSs, and those functionalized with cholesterol after 24 h of incubation. The scale bar corresponds to 100 μm.

**Figure 8:**
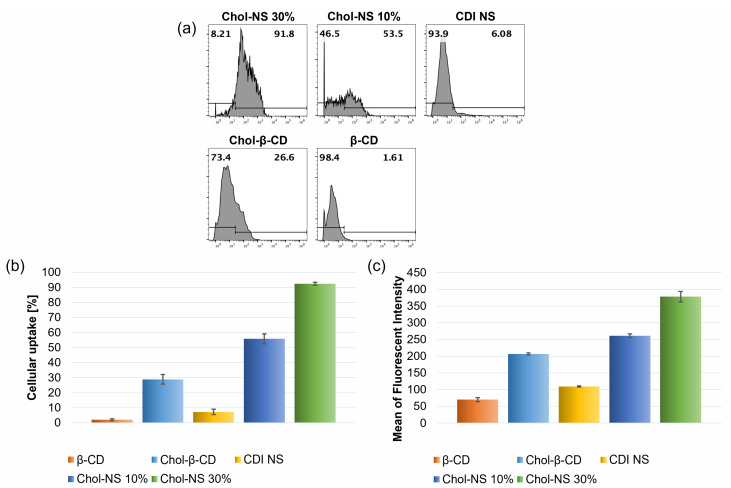
(**a**) Gating results of flow cytometry; (**b**) cellular uptake percentages of β-CD, CDI-NSs, and those functionalized with cholesterol; (**c**) mean of fluorescent intensity.

To investigate this phenomenon, the cellular uptake of blank CD, CDI NSs, and those functionalized with cholesterol at a concentration of 500 µg/mL was evaluated on MCF-7 breast cancer cells. Particles were labeled with fluorophore to allow for the qualitative and quantitative analysis of uptake using fluorescent microscopy and flowcytometry. The fluorescent microscopy results have shown that the cellular uptake of both cholesteryl-functionalized particles was increased, particularly by increasing the cholesterol percentage in Chol-NSs, compared to unfunctionalized particles (Figure 7). Moreover, the quantitative results have demonstrated a remarkably higher uptake for Chol-NSs depending on the content of cholesterol in the structure, up to 95% for Chol-NSs containing 30% Chol-β-CD (Figure 8). The correlation of increased cell internalization based on cholesterol quantity in the carrier structure has been reported in poly(amidoamine) nanoparticles designed for gene delivery, which was mediated via the cholesterol-related endocytosis pathway [20]. Whereas big molecules such as siRNA delivered in the work of Chen et al. [20] required large numbers of cholesteryl moieties in the carrier structure to achieve the enhanced cellular uptake, our material was designed to deliver smaller compounds and showed a significant increase in the cellular internalization even with a relatively small content of cholesterol in the structure of the polymeric carrier.

## 3. Materials and Methods

### 3.1. Materials

Cholesteryl hemisuccinate, 4-(Dimethylamino)pyridine (DMAP), EDC hydrochloride, and 1,1′-carbonyldiimidazole (CDI) were purchased from Sigma Aldrich (Merck; Prague, Czech Republic), β-cyclodextrin was acquired from Cyclolab (Budapest, Hungary), acetone was obtained from Lachner (Neratovice, Czech Republic), and *N*,*N*-Dimethylformamide (DMF) was purchased from Penta (Prague, Czech Republic). Fibroblasts (3T3) and the breast cancer (MCF-7) cell line were purchased from the Pasture Institute National Cell Bank in Tehran (Tehran, Iran), cultivation medium RPMI was obtained from GIBCO Laboratories (ThermoFisher Scientific; Waltham, MA, USA), FBS was acquired from Cegrogen-Biotech (Tehran, Iran), and 3-(4,5-dimethylthiazol-2-yl)2,5-diphenyl tetrazolium bromide (MTT) reagent and resveratrol were purchased from Merck (Tehran, Iran).

### 3.2. Synthesis of Chol-β-CD

The modification of β-CD with cholesterol was based on a previously published method and modified [19]. Cholesteryl hemisuccinate (1.72 g; 3.52 mmol) was dissolved in DMF (60 mL); subsequently, DMAP (0.43 g; 3.52 mmol) and EDC hydrochloride (1.01 g; 5.29 mmol) were added. Then, anhydrous β-CD (2 g; 1.76 mmol) was added in the mixture and the solution was stirred at 45 °C for 24 h. Next, the solution was cooled down to room temperature, precipitated in water (600 mL), filtered, and washed with a small amount of acetone. The resulting powder was then purified with acetone using Soxhlet extractor for 24 h. As a result, Chol-β-CD in the form of white powder (2.83 g; 78%) was obtained. FTIR: ν = 2935–2845 (-CH_3_, -CH_2_), 1735 (-C=O of -COOR). HR-MS: [M + H]^+^ = 1603.73; calc. for C_73_H_119_O_38_^+^ 1603.74. ^13^C SS-NMR (100.52 MHz,): δ (ppm): 171.32, 139.45, 122.26, 112.92, 102.94, 81.17, 72.58, 60.03, 56.64, 49.43, 42.00, 36.63, 31.42, 28.03, 22.24, 18.71, 11.20.

### 3.3. General Procedure of CDI NS Preparation

Carbonyl NSs were prepared by crosslinking CDs with carbonyl diimidazole in a molar ratio of 1:4 (CD:CDI) based on the procedure previously published in the literature [47]. Briefly, anhydrous CD was dissolved in dry DMF, followed by the addition of CDI. The reaction mixture was stirred at 90 °C overnight. The resulting gel was ground and washed with water and acetone. NSs were purified with acetone in a Soxhlet extractor for 24 h to obtain the product in the form of white powder. For the preparation of Chol-NS, the mixtures of Chol-β-CD and native β-CD were used in molar ratios of (Chol-β-CD:β-CD) 1:9 (Chol-NS 10%) and 3:7 (Chol-NS 30%), respectively. The gelation occurred after a few hours; in general, a higher amount of Chol-β-CD increased the period needed for gelation.

**CDI NS:**

Plain nanosponge was prepared from β-CD (2.50 g; 2.20 mmol) and CDI (1.43 g; 8.81 mmol) in anhydrous DMF (15 mL), giving CDI NS (2.59 g; 94%). FTIR: ν = 2936 (-CH_2_), 1746 (-C=O of -COOR). ^13^C ss-NMR (100.52 MHz,) δ 154.19 (C=O), 101.12 (C-1), 80.36 (C-4), 71.37 (C-2, C-3, C-5), and 59.40 (C-6).

**Chol-NS 10%:**

Modified nanosponge was prepared from Chol-β-CD (0.40 g; 0.19 mmol), β-CD (1.97 g; 1.74 mmol), and CDI (1.25 g; 7.72 mmol) in anhydrous DMF (15 mL), resulting in the product Chol-NS 10% (2.45 g; 95%). FTIR: ν = 2936 (-CH_2_), 2867 (-CH_3_), 2849 (-CH_2_), 1746 (-C=O of -COOR), 1702 (-C=O of -COOH), 1374 (C-H from -CH_3_). ^13^C ss-NMR (100.52 MHz,) δ 155.24 (C=O), 135.35 (C=C, cholesterol), 102.16 (C-1), 81.08 (C-4), 72.27 (C-2, C-3, C-5), 60.00 (C-6), and 43.92–16.41 (cholesterol).

**Chol-NS 30%:**

Modified nanosponge was prepared from Chol-β-CD (1.20 g; 0.58 mmol), β-CD (1.53 g; 1.35 mmol), and CDI (1.25 g; 7.72 mmol) in anhydrous DMF (15 mL), resulting in the product Chol-NS 30% (2.71 g; 92%). FTIR: ν = 2936 (-CH_2_), 2867 (-CH_3_), 2849 (-CH_2_), 1746 (-C=O of -COOR), 1702 (-C=O of -COOH), 1374 (C-H from -CH_3_). ^13^C ss-NMR (100,52 MHz,) δ 173.12 (-COO-), 155.24 (C=O), 135.54 (C=C, cholesterol), 102.53 (C-1), 81.83 (C-4), 72.72 (C-2, C-3, C-5), 60.60 (C-6), and 43.92–16.41 (cholesterol).

### 3.4. Modification of Chol-NS with Fluorescent Moiety

The labeling procedure was based on a previously published method [4] with minor modifications. Briefly, cyclodextrin nanosponges (0.5 g) were suspended in DMSO (2 mL) and stirred for 30 min at 45 °C. Subsequently, the fluorescein (50 mg) and CDI (25 mg) were added to the suspension. The mixture was stirred at 45 °C for an additional 2 h. The product was washed several times with acetone to remove DMF and unreacted dyes and finally extracted with acetone using pressurized solvent extraction.

### 3.5. Preparation of Chol-NS Suspensions and Loading with Resveratrol

Nanosponge particles were suspended in distilled water at a concentration of 1 mg/mL and stirred for 1 h. Subsequently, the suspensions were sonicated for 15–20 min to eliminate any aggregates. The formulations of resveratrol loaded into CD and nanosponges (RSV + CD or RSV + NS), respectively, were prepared at a ratio of 1:4 (*w*/*w*; RSV:CD/NSs). The suspensions were agitated for 30 min at room temperature, and they were filtered with a 0.45 µm syringe filter before the cells’ treatment.

### 3.6. Characterization

#### 3.6.1. FTIR Analysis

Fourier-transform infrared analysis (FTIR) was carried out using Nicolet iZ10 by ThermoFisher Scientific (Waltham, MA, USA) with diamond crystal. The ATR technique was used for obtaining the spectra, averaging 8 scans of each; the background and sample were in the range of 4000–400 cm^−1^ with a resolution of 4 cm^−1^. The spectra were evaluated using the Omnic software (v9).

#### 3.6.2. FIA-HRMS Analysis

Flow Injection Analysis (FIA) involves the injection of the sample into a continuous solvent carrier flow directly connected to the ESI source of the MS instrument without any prior chromatographic separation. The sample was prepared at a concentration of 0.1 mg/mL in MeOH. The diluted sample was injected into the flowing solvent (MeOH:H_2_O, 20:80 (*v*/*v*)) acidified with 0.1% (*v*/*v*) formic acid at a flow rate of 300 μL/min and delivered directly to the ESI source by a syringe pump at constant flow (20 μL/min).

The HRMS analysis was performed using an Orbitrap IQ-X mass spectrometer (Thermo Fisher Scientific; San Jose, CA, USA) equipped with a Heated ElectroSpray Ionization (HESI) source. The MS parameters were optimized to enhance ionization and to minimize in-source fragmentation. The ion source was operated in positive-ion mode using the following settings: spray voltage of 2500 V (+), ion transfer tube temperature of 275 °C, and vaporizer temperature of 290 °C. Nitrogen was used as both a sheath gas and auxiliary gas, with flow rates set at 40 and 10 arbitrary units (a.u.), respectively. The mass spectra were recorded in the *m*/*z* range of 100–2000 at a target resolution of 120,000 (FWHM at 200 *m*/*z*). The mass error was less than 3 ppm (part per million). For structural confirmation of the compound, tandem mass spectrometry experiments (MS^2^) were performed both by traditional collision-induced dissociation (CID) and higher energy collisional dissociation (HCD) at a target resolution of 30,000 (FWHM at 200 *m*/*z*). The CID and HCD techniques offer diverse fragmentation paths, ensuring accurate structural characterization when used together. Various normalized collision energies (NCE) ranging from 25 to 30 were examined. All data were processed by FreeStyle software (v1.8 SP2, Thermo Fisher Scientific; San Jose, CA, USA).

#### 3.6.3. Solid-State Nuclear Magnetic Resonance (NMR)

The solid-state NMR spectra were recorded using the NMR spectrometer JNM-ECZ400R/M1 (400 MHz, JEOL, Tokyo, Japan) at ambient temperature. Samples were packed into 3.2 mm ZrO_2_ rotors, with all ^13^C (at a Larmor frequency of 100.53 MHz) spectra recorded using direct pulse excitation at an MAS of 20 kHz with high-powered (TPPM) ^1^H decoupling, externally referenced to γ-glycine (174.1 ppm carbonyl signal). The length of the π/2 pulse was 2.13 µs at 8.7 dB. Relaxation delays and number of scans were optimized based on individual samples.

#### 3.6.4. Dynamic Light Scattering (DLS)

Particle size measurement was performed on a 90-plus particle sizer (Malvern Instruments, Malvern, UK). The suspensions were prepared according to the previously described protocol to measure their size (represented by the hydrodynamic diameter) and polydispersity index (PdI). Measurements were performed at room temperature in disposable cuvettes. Each measurement was performed in triplicate.

#### 3.6.5. Drug Loading and Encapsulation Efficiency

The quantification of loaded resveratrol and assessment of encapsulation efficiency were based on a method previously published by [47]. The loaded nanosponges were dispersed in ethanol (c = 2.5 mg/mL) and sonicated for 1 h. Obtained extracts were filtered and analyzed with HPLC.

The HPLC analysis was conducted using an HPLC system (PerkinElmer, Waltham, MA, USA) with a UV detector (Flexar UV/Vis LC spectrophotometer) and C18 analytical column (4.6 mm × 250 mm, 5 μm). The mobile phase containing methanol and water (52:48, *v/v*) was adjusted by acetic acid (0.5% in water). The elution was executed at room temperature with a flow rate of 1 mL/min, and the detection was completed at a wavelength of 305 nm.

The calculations of drug loading capacity and encapsulation efficiency were based on Equations (1) and (2), respectively.

(1) $$Loading\;capacity\left[\%\right]=\frac{m_{loaded\;drug}}{m_{loaded\;NS}}\times 100$$

(2) $$Encapsulation\;efficiency\left[\%\right]=\frac{m_{loaded\;NS}\cdot loading\;capacity}{m_{total\;drug}}\times 100$$

#### 3.6.6. Cell Culturing Conditions

Fibroblast (3T3) and MCF-7 cells were sourced from the Pasteur Institute National Cell Bank of Tehran, Iran. The cells were cultured in RPMI 1640 medium supplemented with 10% FBS and 1% penicillin/streptomycin solution. The flask was placed in a humidified incubator set to 37 °C with 5% CO_2_. The medium was replaced every two days, and the cells were detached and collected after trypsinization once they reached 80–90% confluence in each passage.

#### 3.6.7. In Vitro Cytotoxicity

The 3-(4,5-dimethylthiazol-2-yl)-2,5-diphenyl tetrazolium bromide (MTT) assay was utilized to assess the in vitro cell viability. Fibroblast and MCF-7 cells were seeded in 96-well plates and incubated until they reached 80% confluence, after which they were exposed to the synthesized materials at specific concentrations. Vehicle control groups were also included. After the exposure, the growth medium was removed, and the cells were gently rinsed with 1X phosphate-buffered saline (PBS). Fresh serum-free medium (100 µL) containing 0.5 mg/mL MTT solution was added, and the cells were incubated for 4 h at 37 °C. Subsequently, the MTT solution was removed and replaced with 100 µL dimethyl sulfoxide (DMSO). After an additional hour of incubation at 37 °C, cell viability was measured using a microplate ELISA reader (Biotech, San Jose, CA, USA, Gen5™ software, v3.16.10). Absorbance was recorded at 560 nm, with the background measured at 630 nm, using DMSO as a blank. Background and blank values were subtracted, and cell viability was expressed as a percentage relative to the untreated control, which was considered 100% viable. The viability was calculated based on the optical density (OD) values of the treated and untreated cells, as outlined in Equation (3).

(3) $$Cell\;viability\left[\%\right]=\frac{{OD}_{treated}-{OD}_{blank}}{{OD}_{untreated}-{OD}_{blank}}\times 100$$

#### 3.6.8. Cellular Uptake

To check the cellular uptake, the MCF-7 cell line was exposed to formulations at a concentration 500 µg/mL. Particles were labeled with fluorophore and the results were analyzed both qualitatively and quantitatively. For the qualitative analysis, the cancer cells were seeded in a 6-well plate at a density of 5 × 10^5^ cell/well, incubated overnight, and then exposed to particles for 24 h. Thereafter, the cells were examined by the Cytation 5 Cell Imaging Multi-Mode Reader (San Jose, CA USA; Gen5™ software, v3.16.10). The quantitative cellular uptake of particles in MCF-7 cancer cells was assessed using flow cytometry. The cells at a density of 20 × 10^4^ cell/well were seeded in a 12-well plate and incubated for 24 h to obtain an 80% confluency. The cells were treated with fluorophore-labeled particles for 24 h at 37 °C and 5% CO_2_. Subsequently, the cells were trypsinized and washed with PBS to perform the analysis of the particle uptake potential. The examination was based on the fluorescent intensity, and it was carried out using a fluorescence-activated cell sorting (FACS) caliber flow cytometer (BD Pharmigen™, BD Biosciences, San Jose, CA, USA).

#### 3.6.9. Statistical Analysis

The results of the in vitro cytotoxicity and cellular uptake experiments were analyzed using a one-way ANOVA. In the figures, error bars represent the standard deviation, and significance determined using a one-way ANOVA is demonstrated (ns, not significant; * *p* < 0.05; ** *p* < 0.01; *** *p* < 0.001; **** *p* < 0.0001).

## 4. Conclusions

Cyclodextrin nanosponges functionalized with cholesterol were prepared via a synthetic method that allows for a more controlled process compared to simple polymer postmodification. As a result, materials with different contents of cholesterol moieties in the structure were manufactured, and the effect of functionalization on the treatment efficacy and cellular uptake was investigated. The results showed that cholesteryl-functionalized nanosponges significantly increased the anticancer activity of resveratrol. This natural antioxidant has poor bioavailability in its free form, which is limiting its therapeutic potential. Moreover, the flow cytometry results showed that the functionalization of material plays a critical role in the internalization into cancer cells, increasing the cellular uptake severalfold. Therefore, cholesterol-functionalized cyclodextrin nanosponges proved to be a promising material for the delivery of anticancer drugs and natural active compounds with poor aqueous solubility.

## Figures and Tables

**Table 1:** Hydrodynamic diameter of the synthesized Chol-NS and CDI NS.

	Hydrodynamic Diameter [nm]	PDI
Chol-NS 10%	195 ± 3	0.26 ± 0.02
Chol-NS 30%	168 ± 18	0.34 ± 0.03
CDI NS	211 ± 6	0.25 ± 0.01

**Table 2:** Loading capacity and encapsulation efficiency of Chol-NS and CDI NS loaded with resveratrol.

	Loading Capacity [%]	Encapsulation Efficiency [%]
Chol-NS 10%	19	76
Chol-NS 30%	12	46
CDI NS	13	50

## Data Availability

The data are available upon request from the authors.

## References

[1] Crini G. (2014). Review: A History of Cyclodextrins. Chem. Rev..

[2] Ioele G., De Luca M., Garofalo A., Ragno G. (2017). Photosensitive Drugs: A Review on Their Photoprotection by Liposomes and Cyclodextrins. Drug Deliv..

[3] Mousazadeh H., Pilehvar-Soltanahmadi Y., Dadashpour M., Zarghami N. (2021). Cyclodextrin Based Natural Nanostructured Carbohydrate Polymers as Effective Non-Viral siRNA Delivery Systems for Cancer Gene Therapy. J. Control. Release.

[4] Rubin Pedrazzo A., Caldera F., Zanetti M., Appleton S.L., Dhakar N.K., Trotta F. (2020). Mechanochemical Green Synthesis of Hyper-Crosslinked Cyclodextrin Polymers. Beilstein J. Org. Chem..

[5] Wang J., Yang X.-Q., Li N., Wang L.-L., Xu X.-Y., Zhang C. (2023). A Cyclodextrin-Based Turn-off Fluorescent Probe for Naked-Eye Detection of Copper Ions in Aqueous Solution. Spectrochim. Acta A Mol. Biomol. Spectrosc..

[6] Khazaei Monfared Y., Mahmoudian M., Caldera F., Pedrazzo A.R., Zakeri-Milani P., Matencio A., Trotta F. (2023). Nisin Delivery by Nanosponges Increases Its Anticancer Activity against In-Vivo Melanoma Model. J. Drug Deliv. Sci. Technol..

[7] Khazaei Monfared Y., Mahmoudian M., Cecone C., Caldera F., Zakeri-Milani P., Matencio A., Trotta F. (2022). Stabilization and Anticancer Enhancing Activity of the Peptide Nisin by Cyclodextrin-Based Nanosponges against Colon and Breast Cancer Cells. Polymers.

[8] Monfared Y.K., Pedrazzo A.R., Mahmoudian M., Caldera F., Zakeri-Milani P., Valizadeh H., Cavalli R., Matencio A., Trotta F. (2023). Oral Supplementation of Solvent-Free Kynurenic Acid/Cyclodextrin Nanosponges Complexes Increased Its Bioavailability. Colloids Surf. B Biointerfaces.

[9] Daga M., Ullio C., Argenziano M., Dianzani C., Cavalli R., Trotta F., Ferretti C., Zara G.P., Gigliotti C.L., Ciamporcero E.S. (2016). GSH-Targeted Nanosponges Increase Doxorubicin-Induced Toxicity “in Vitro” and “in Vivo” in Cancer Cells with High Antioxidant Defenses. Free Radic. Biol. Med..

[10] Rao M.R.P., Chaudhari J., Trotta F., Caldera F. (2018). Investigation of Cyclodextrin-Based Nanosponges for Solubility and Bioavailability Enhancement of Rilpivirine. Aaps Pharmscitech.

[11] Trotta F., Cavalli R. (2009). Characterization and Applications of New Hyper-Cross-Linked Cyclodextrins. Compos. Interfaces.

[12] Mognetti B., Barberis A., Marino S., Berta G., Francia S., Trotta F., Cavalli R. (2012). In Vitro Enhancement of Anticancer Activity of Paclitaxel by a Cremophor Free Cyclodextrin-Based Nanosponge Formulation. J. Incl. Phenom. Macrocycl. Chem..

[13] Trotta F., Dianzani C., Caldera F., Mognetti B., Cavalli R. (2014). The Application of Nanosponges to Cancer Drug Delivery. Expert Opin. Drug Deliv..

[14] Olteanu A.A., Aramă C.-C., Radu C., Mihăescu C., Monciu C.-M. (2014). Effect of β-Cyclodextrins Based Nanosponges on the Solubility of Lipophilic Pharmacological Active Substances (Repaglinide). J. Incl. Phenom. Macrocycl. Chem..

[15] Rao M.R.P., Shirsath C. (2017). Enhancement of Bioavailability of Non-Nucleoside Reverse Transciptase Inhibitor Using Nanosponges. AAPS PharmSciTech.

[16] Zainuddin R., Zaheer Z., Sangshetti J.N., Momin M. (2017). Enhancement of Oral Bioavailability of Anti-HIV Drug Rilpivirine HCl through Nanosponge Formulation. Drug Dev. Ind. Pharm..

[17] Olim F., Neves A.R., Vieira M., Tomás H., Sheng R. (2021). Self-Assembly of Cholesterol-Doxorubicin and TPGS into Prodrug-Based Nanoparticles with Enhanced Cellular Uptake and Lysosome-Dependent Pathway in Breast Cancer Cells. Eur. J. Lipid Sci. Technol..

[18] Misiak P., Niemirowicz-Laskowska K., Markiewicz K.H., Wielgat P., Kurowska I., Czarnomysy R., Misztalewska-Turkowicz I., Car H., Bielawski K., Wilczewska A.Z. (2023). Doxorubicin-Loaded Polymeric Nanoparticles Containing Ketoester-Based Block and Cholesterol Moiety as Specific Vehicles to Fight Estrogen-Dependent Breast Cancer. Cancer Nanotechnol..

[19] Singh P., Ren X., Guo T., Wu L., Shakya S., He Y., Wang C., Maharjan A., Singh V., Zhang J. (2018). Biofunctionalization of β-Cyclodextrin Nanosponges Using Cholesterol. Carbohydr. Polym..

[20] Chen C.-J., Wang J.-C., Zhao E.-Y., Gao L.-Y., Feng Q., Liu X.-Y., Zhao Z.-X., Ma X.-F., Hou W.-J., Zhang L.-R. (2013). Self-Assembly Cationic Nanoparticles Based on Cholesterol-Grafted Bioreducible Poly(Amidoamine) for siRNA Delivery. Biomaterials.

[21] Kim W.J., Chang C.-W., Lee M., Kim S.W. (2007). Efficient siRNA Delivery Using Water Soluble Lipopolymer for Anti-Angiogenic Gene Therapy. J. Control. Release.

[22] Misiak P., Niemirowicz-Laskowska K., Markiewicz K.H., Misztalewska-Turkowicz I., Wielgat P., Kurowska I., Siemiaszko G., Destarac M., Car H., Wilczewska A.Z. (2020). Evaluation of Cytotoxic Effect of Cholesterol End-Capped Poly(N-Isopropylacrylamide)s on Selected Normal and Neoplastic Cells. Int. J. Nanomed..

[23] Auzély-Velty R., Djedaïni-Pilard F., Désert S., Perly B., Zemb T. (2000). Micellization of Hydrophobically Modified Cyclodextrins. 1. Micellar Structure. Langmuir.

[24] Klaus A., Fajolles C., Bauer M., Collot M., Mallet J.-M., Daillant J. (2011). Amphiphilic Behavior and Membrane Solubility of a Dicholesteryl-Cyclodextrin. Langmuir.

[25] Wang T., Chipot C., Shao X., Cai W. (2011). Structural Characterization of Micelles Formed of Cholesteryl-Functionalized Cyclodextrins. Langmuir.

[26] Singh V., Guo T., Xu H., Wu L., Gu J., Wu C., Gref R., Zhang J. (2017). Moisture Resistant and Biofriendly CD-MOF Nanoparticles Obtained via Cholesterol Shielding. Chem. Commun..

[27] López-Valverde N., López-Valverde A., Montero J., Rodríguez C., Macedo de Sousa B., Aragoneses J.M. (2023). Antioxidant, Anti-Inflammatory and Antimicrobial Activity of Natural Products in Periodontal Disease: A Comprehensive Review. Front. Bioeng. Biotechnol..

[28] Larik M.O., Ahmed A., Khan L., Iftekhar M.A. (2023). Effects of Resveratrol on Polycystic Ovarian Syndrome: A Systematic Review and Meta-Analysis of Randomized Controlled Trials. Endocrine.

[29] Yang Q., Meng D., Zhang Q., Wang J. (2024). Advances in the Role of Resveratrol and Its Mechanism of Action in Common Gynecological Tumors. Front. Pharmacol..

[30] Xu X.-L., Deng S.-L., Lian Z.-X., Yu K. (2021). Resveratrol Targets a Variety of Oncogenic and Oncosuppressive Signaling for Ovarian Cancer Prevention and Treatment. Antioxidants.

[31] Tang H., Wen J., Qin T., Chen Y., Huang J., Yang Q., Jiang P., Wang L., Zhao Y., Yang Q. (2023). New Insights into Sirt1: Potential Therapeutic Targets for the Treatment of Cerebral Ischemic Stroke. Front. Cell. Neurosci..

[32] Chandrasekaran V., Hediyal T.A., Anand N., Kendaganna P.H., Gorantla V.R., Mahalakshmi A.M., Ghanekar R.K., Yang J., Sakharkar M.K., Chidambaram S.B. (2023). Polyphenols, Autophagy and Neurodegenerative Diseases: A Review. Biomolecules.

[33] Guo S., Chen M., Li S., Geng Z., Jin Y., Liu D. (2023). Natural Products Treat Colorectal Cancer by Regulating miRNA. Pharmaceuticals.

[34] Roshani M., Jafari A., Loghman A., Sheida A.H., Taghavi T., Tamehri Zadeh S.S., Hamblin M.R., Homayounfal M., Mirzaei H. (2022). Applications of Resveratrol in the Treatment of Gastrointestinal Cancer. Biomed. Pharmacother..

[35] Badawi J.K. (2023). Resveratrol Used as Nanotherapeutic: A Promising Additional Therapeutic Tool against Hormone-Sensitive, Hormone-Insensitive and Resistant Prostate Cancer. Am. J. Clin. Exp. Urol..

[36] Ahmed H., Abdelraheem A., Salem M., Sabry M., Fekry N., Mohamed F., Saber A., Piatti D., Sabry M., Sabry O. (2023). Suppression of Breast Cancer: Modulation of Estrogen Receptor and Downregulation of Gene Expression Using Natural Products. Nat. Prod. Res..

[37] Alaouna M., Penny C., Hull R., Molefi T., Chauke-Malinga N., Khanyile R., Makgoka M., Bida M., Dlamini Z. (2023). Overcoming the Challenges of Phytochemicals in Triple Negative Breast Cancer Therapy: The Path Forward. Plants.

[38] Kowsari H., Davoodvandi A., Dashti F., Mirazimi S.M.A., Bahabadi Z.R., Aschner M., Sahebkar A., Gilasi H.R., Hamblin M.R., Mirzaei H. (2023). Resveratrol in Cancer Treatment with a Focus on Breast Cancer. Curr. Mol. Pharmacol..

[39] Choi I., Li N., Zhong Q. (2022). Enhancing Bioaccessibility of Resveratrol by Loading in Natural Porous Starch Microparticles. Int. J. Biol. Macromol..

[40] Yi J., He Q., Peng G., Fan Y. (2022). Improved Water Solubility, Chemical Stability, Antioxidant and Anticancer Activity of Resveratrol via Nanoencapsulation with Pea Protein Nanofibrils. Food Chem..

[41] Gadag S., Narayan R., Nayak A.S., Catalina Ardila D., Sant S., Nayak Y., Garg S., Nayak U.Y. (2021). Development and Preclinical Evaluation of Microneedle-Assisted Resveratrol Loaded Nanostructured Lipid Carriers for Localized Delivery to Breast Cancer Therapy. Int. J. Pharm..

[42] Lee D.G., Lee M., Go E.B., Chung N. (2022). Resveratrol-Loaded Gold Nanoparticles Enhance Caspase-Mediated Apoptosis in PANC-1 Pancreatic Cells via Mitochondrial Intrinsic Apoptotic Pathway. Cancer Nanotechnol..

[43] Thipe V.C., Amiri K.P., Bloebaum P., Karikachery A.R., Khoobchandani M., Katti K.K., Jurisson S.S., Katti K.V. (2019). Development of Resveratrol-Conjugated Gold Nanoparticles: Interrelationship of Increased Resveratrol Corona on Anti-Tumor Efficacy against Breast, Pancreatic and Prostate Cancers. Int. J. Nanomed..

[44] Ioniţă S., Lincu D., Mitran R.-A., Ziko L., Sedky N.K., Deaconu M., Brezoiu A.-M., Matei C., Berger D. (2022). Resveratrol Encapsulation and Release from Pristine and Functionalized Mesoporous Silica Carriers. Pharmaceutics.

[45] Marinheiro D., Ferreira B.J.M.L., Oskoei P., Oliveira H., Daniel-da-Silva A.L. (2021). Encapsulation and Enhanced Release of Resveratrol from Mesoporous Silica Nanoparticles for Melanoma Therapy. Materials.

[46] Das S., Lin H.-S., Ho P.C., Ng K.-Y. (2008). The Impact of Aqueous Solubility and Dose on the Pharmacokinetic Profiles of Resveratrol. Pharm. Res..

[47] Dhakar N.K., Matencio A., Caldera F., Argenziano M., Cavalli R., Dianzani C., Zanetti M., Manuel Lopez-Nicolas J., Trotta F. (2019). Comparative Evaluation of Solubility, Cytotoxicity and Photostability Studies of Resveratrol and Oxyresveratrol Loaded Nanosponges. Pharmaceutics.

[48] Shi Y., Zhou J., Jiang B., Miao M. (2017). Resveratrol and Inflammatory Bowel Disease. Ann. N. Y. Acad. Sci..

[49] Gruber A., Navarro L., Klinger D. (2020). Reactive Precursor Particles as Synthetic Platform for the Generation of Functional Nanoparticles, Nanogels, and Microgels. Adv. Mater. Interfaces.

[50] Lee A.L.Z., Venkataraman S., Sirat S.B.M., Gao S., Hedrick J.L., Yang Y.Y. (2012). The Use of Cholesterol-Containing Biodegradable Block Copolymers to Exploit Hydrophobic Interactions for the Delivery of Anticancer Drugs. Biomaterials.

[51] Qin Y., Peng X. (2020). Synthesis of Biocompatible Cholesteryl–Carboxymethyl Xylan Micelles for Tumor-Targeting Intracellular DOX Delivery. ACS Biomater. Sci. Eng..

[52] Muddineti O.S., Kumari P., Ray E., Ghosh B., Biswas S. (2017). Curcumin-Loaded Chitosan–Cholesterol Micelles: Evaluation in Monolayers and 3D Cancer Spheroid Model. Nanomedicine.

[53] Shaikh V.A.E., Lonikar S.V., Dhobale D.A., Pawar G.M. (2007). Cholesterol-Linked β-Cyclodextrin—A Thermotropic Liquid-Crystalline Derivative. Bull. Chem. Soc. Jpn..

[54] Mok Z.H. (2024). The Effect of Particle Size on Drug Bioavailability in Various Parts of the Body. Pharm. Sci. Adv..

[55] Bommana M.M., Raut S. (2018). Brain Targeting of Payload Using Mild Magnetic Field. Nanostructures for the Engineering of Cells, Tissues and Organs.

[56] Tang L., Yang X., Yin Q., Cai K., Wang H., Chaudhury I., Yao C., Zhou Q., Kwon M., Hartman J.A. (2014). Investigating the Optimal Size of Anticancer Nanomedicine. Proc. Natl. Acad. Sci. USA.

[57] Krabicová I., Appleton S.L., Tannous M., Hoti G., Caldera F., Rubin Pedrazzo A., Cecone C., Cavalli R., Trotta F. (2020). History of Cyclodextrin Nanosponges. Polymers.

[58] Hao X., Sun X., Zhu H., Xie L., Wang X., Jiang N., Fu P., Sang M. (2021). Hydroxypropyl-β-Cyclodextrin-Complexed Resveratrol Enhanced Antitumor Activity in a Cervical Cancer Model: In Vivo Analysis. Front. Pharmacol..

[59] Zhang W., Zhang R., Chang Z., Wang X. (2022). Resveratrol Activates CD8+ T Cells through IL-18 Bystander Activation in Lung Adenocarcinoma. Front. Pharmacol..

[60] Ansari K.A., Vavia P.R., Trotta F., Cavalli R. (2011). Cyclodextrin-Based Nanosponges for Delivery of Resveratrol: In Vitro Characterisation, Stability, Cytotoxicity and Permeation Study. AAPS PharmSciTech.

[61] Misiak P., Markiewicz K.H., Szymczuk D., Wilczewska A.Z. (2020). Polymeric Drug Delivery Systems Bearing Cholesterol Moieties: A Review. Polymers.

